# Preliminary study on discrimination of transgenic cotton seeds using terahertz time‐domain spectroscopy

**DOI:** 10.1002/fsn3.1846

**Published:** 2020-08-27

**Authors:** Bin Li, Xiaochen Shen

**Affiliations:** ^1^ Beijing Research Center for Information Technology in Agriculture Beijing China

**Keywords:** cotton seeds, discrimination, terahertz spectroscopy, transgenic and nontransgenic DNA

## Abstract

Presence of genetically modified (GM) organisms is considered to be controversial by legislation and public. It is very important to develop detection methods for early discriminations. Conventional gene detection methods, including protein detection (PCR, ELISA, and so on) and DNA detection (Southern blot, GC/MS, and so on), have the disadvantages of high costs, time‐consuming, complex operations, and destructive of the samples. Terahertz spectroscopy (THz) is a brand‐new radiation with many unique advantages. Most biological macromolecules have fingerprint characteristics in THz band from the current recognition. In this study, feasibility of identifying the transgenic cotton seeds from nontransgenic counterparts using THz spectroscopy method was investigated. The transgenic cotton seeds‐Lumianyan No.28 and nontransgenic cotton seeds‐Xinluzao No.51 were selected and the sample‐making methods were studied; then the refractive and absorption curves of samples were got and given a detailed discussion; finally, absorption index of transgenic and nontransgenic DNA was observed and discussed. The results showed there were small fluctuations in THz band, and refractive index of transgenic seeds was lower than nontransgenic ones and had obvious turning point at 1.4–2.0 THz region. There were significant peaks in 1.0–1.2 and 1.3–1.5 THz regions for the transgenic cotton seeds. Transgenic DNA had higher absorption index than nontransgenic DNA, and there were 3–4 peaks corresponding to the cotton seed samples in 1.0–1.6 THz region. These results showed cotton seeds samples can provide important bio‐information in THz band, and this study provided a basis for developing potential THz‐based gene detection technologies.

## Graphical abstract

In this paper, the authors conducted experiment to obtain and observe absorption differences of the transgenic/nontransgenic cotton seeds and DNA in terahertz spectrum band and tried to give the effects and mechanism interpretation of THz spectroscopy on transgenic organisms and gene segments, which provided a basis for developing potential THz‐based gene detection technologies.

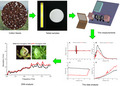

## 1 INTRODUCTION

Nowadays, bio‐technology's quick development has brought a great variety of transgenes transferred into crops and products‐derived varieties (Lois, Hong, Pease, Brown, & Baltimore, 2002; Zhao, Qian, Wang, & Huang, 2007). Though genetically modified (GM) organisms may have the advantages of high yield and insect resistance, it is found that not all of the global markets fully compliant with GM products, which may introduce new allergens, develop resistant bacterial strains and alter environmental biodiversity, causing biosafety issues (Bakshi, 2003).

In recent years, cotton genetic transformation technologies have made great progress and cultivation of transgenic cotton is increasing sharply (Ali et al., 2016; Aragao, Vianna, Carvalheira, & Rech, 2005). To identify the presence of GM organisms in plant and products derived, many detection methods have been developed. The conventional gene distinguish methods refer to DNA detection (GC/MS, Southern blot, and so on) and protein detection (ELISA, PCR, Western blot, and so on) (Leimanis et al., 2006; Nesvold, Kristoffersen, Holst‐Jensen, & Berdal, 2005; Vaïtilingom, Pijnenburg, Gendre, & Brignon, 1999), which can provide accurate detection results. However, the conventional methods have many disadvantages of time‐consuming, expensive equipment, complex operations, and destructive of the samples, difficult to fulfill current demands. Existing researches have proved that the above objectives could be achieved by spectral methods such as near‐infrared, Raman, hyperspectral imaging, and visible near‐infrared (Baranski & Baranska, 2008; Xie, Ying, Ying, Yu, & Fu, 2007; Xu, Liu, Xie, & Ying, 2014). Such methods own the advantages of nondestructive monitor, fast and simple operation. As a brand‐new technique developed in recent years, terahertz (THz) spectroscopy is an electromagnetic wave of 0.1–10 THz (30 μm–3 mm in wavelength) in frequency. Most biological macromolecules (protein, DNA, and amino acids) have fingerprint characteristics in THz band from the current recognition (Tonouchi, 2007), which means THz spectroscopy can provide important information for detecting and distinguishing biological samples (Fischer, Walther, & Jepsen, 2002; Jiao, Si, Li, Zhang, & Xu, 2010; Liu, Liu, Hu, Yang, & Zheng, 2016). However, complex intramolecular and intermolecular characteristics of bio‐polymers and polyethylene, such as skeletal vibrations or hydrogen bonding, are affected by each other and have little been known until the THz spectroscopy and imaging technique comes and, the sample‐making technologies for the transgenic and nontransgenic cotton seeds to get useful signals are worth studying. In the previous study (Xu, Li, Zuo, & Zhang, 2015), it was found that, due to different sizes of samples’ particles, there were some differences in the vibration peak intensity, peak position, and peak numbers in the absorption spectra. And the scattering effect should be responsible for this. Due to the Mie scatting, it is worthy to note that when the particle size of sample was smaller, the absorption peak strength was more obvious, and predominant repeatability of THz spectra was better (Xu et al., 2015), which should be considered in this research. In addition, polyethylene (PE) is one of the commonly selected polymer plastics for sample‐making material in THz technologies in current research (Wietzke et al., 2010), which provides a good sample‐making method for this research. Considering the above, the objective of this research was to obtain and observe the absorption difference between the transgenic and nontransgenic cotton seeds in THz band and try to explore a method to identify the transgenic cotton seeds from its counterparts using THz spectroscopy probing methods. The results will be helpful to understand the mechanism of the risk from transgenic organisms to cotton seeds not only for biotechnological research but also for the agricultural production.

## 2 MATERIALS AND METHODS

### 2.1 Cotton seeds samples

The transgenic and nontransgenic cotton seeds were provided by Beijing Academy of Agriculture and Forestry Sciences. In the present study, two classes (transgenic and nontransgenic cotton seeds) were defined. The information of cotton seeds samples is shown in Table 1.

**Table 1:** The parameters for sample preparation in different treatments

Items	Transgenic/nontransgenic cotton seeds	Polyethylene
I	+	+ (Interlayer)
II	+	+ (Mixing)
III	+	—

Lumianyan No.28 (LMY‐1) is transgenic cotton seed, and Xinluzao No.51 (XLZ) is nontransgenic cotton seed. The transgenic variety is pest‐resistant cotton and the non‐transgenic one is without it. The samples of cotton seed were stored in sealed plastic bags at 4℃ until use.

### 2.2 Sample preparation and experimental design

Because there is more lipid content in cotton seeds, standard polyethylene chemical powder, which has no strong absorption features within the THz spectrum, was chosen firstly to be the medium. The tough testa of all the transgenic and nontransgenic cotton seeds was removed firstly.

In this experiment, sizes of the cotton seeds’ particles were grinded at 80 nm in Nano‐scale. Room temperature grinding of seed powder was conducted by a planetary ball mill (YXQM‐1L) using media tablets of the nanoparticle scale. In order to explore sample‐making method and data acquisition method in THz band, the current research was composed of three experiments:

Experiment‐1 was to optimize THz spectroscopy technology and was divided into three controlled groups according to with/without polyethylene disturbance (Table 1).

Group‐I: Polyethylene chemical powder was used as the interlayer of the tablet.
Group‐II: Polyethylene chemical powder was mixed in the seed powder and made for the tablet samples.
Group‐III: Only transgenic/non‐transgenic cotton seeds were made for the samples without any polyethylene chemicals.

Experiment‐2 was to investigate the viability and effectiveness of THz spectroscopy for discrimination of transgenic (LMY‐1) and nontransgenic cotton seeds (XLZ).

Experiment‐3 was to explore the mechanism of THz spectroscopy for discrimination of transgenic and non‐transgenic organisms by using molecular DNA segments.

**Figure 1:**
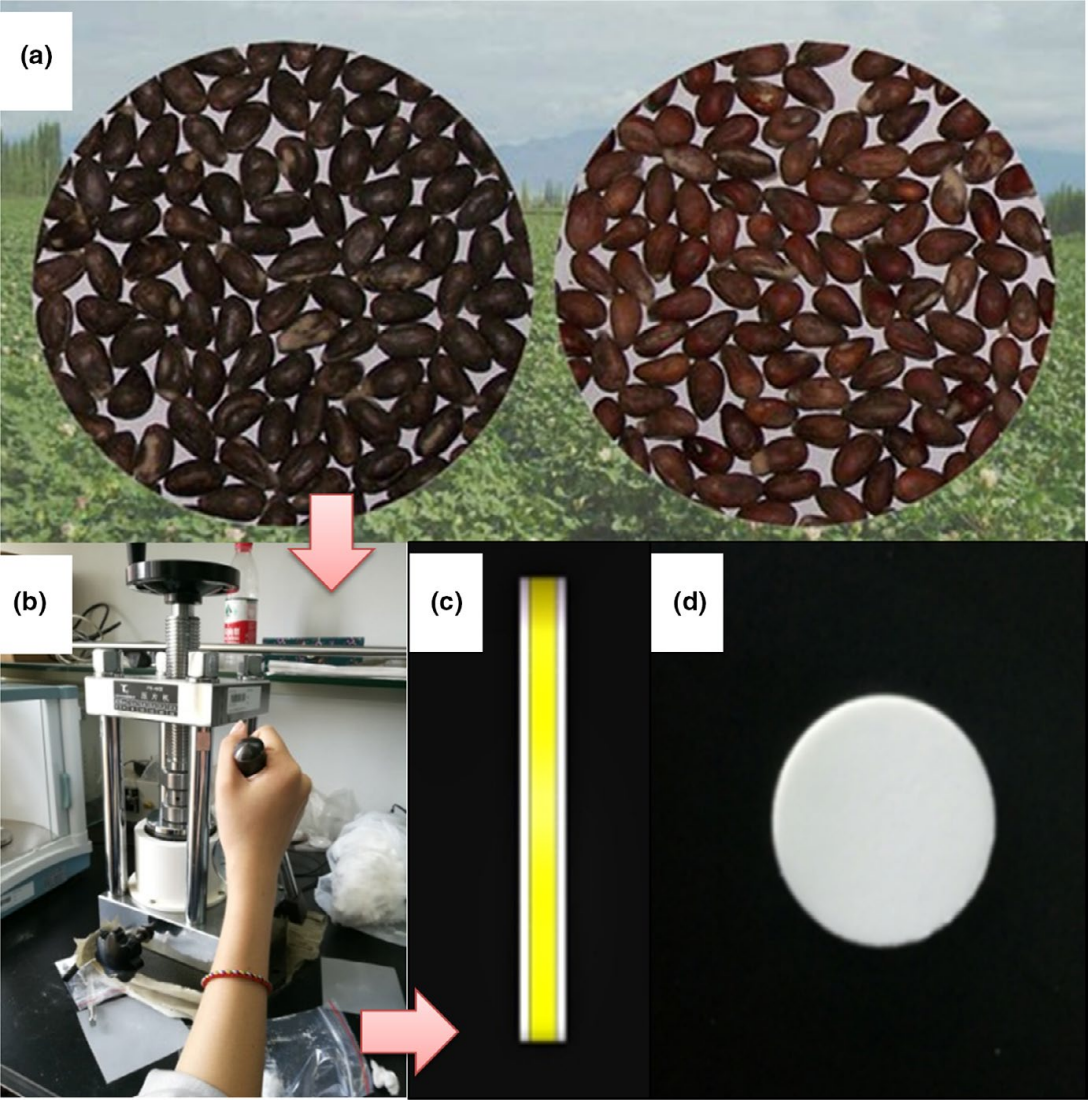
The transgenic and nontransgenic cotton seeds (a), pressure device (b) and the side (c)/front (d) view of the prepared samples

All tablet samples were made into 1 mm thickness sheets. The diameter of each plate was 13 mm in size. The main procedures of this research were shown in Figure 1.

### 2.3 Experimental setup

All reflection and transmission experiments were performed in laboratory atmosphere at room temperature on a Menlo Systems TERA K15, THz time‐domain spectroscopy system with fiber coupling (And & Schmuttenmaer, 1996). The central wavelength of the THz generator and detector is 1,550 nm and the optical power is 33 mW. The TERA K15 was allowed for the tight focusing of the THz beam down to approximately 1 mm in diameter via four plano‐convex lenses. Measuring the reflection or transmission spectrum by a dedicated special rotating table that rotates the detector and associated lens together to the appropriate angle of incidence required for the sample direction. A sample holder was then mounted on top of the pivoting point of this mechanism. The samples were held in place in this holder tightly, to ensure that each sample was mounted as flat as possible and that avoid moving the device to the greatest extent when exchanging samples to reduce errors. During the test, in order to avoid THz pulse being absorbed by water molecules in the air, nitrogen should be filled in the sealed experimental device so that the temperature in the sealed device is about 21℃ and the relative humidity is <5% (Li, Li, & Ye, 2020).

The THz beam was focused onto the sample by the paraboloids and reflected by the sample layers. The reflection beam, preserving the phase and amplitude information of the sample, was collected and sent to optically gated THz dipole detector. The reflection signal was amplified and processed on the flight for each pixel to obtain the THz data (time‐domain data) that can be transformed into frequency‐domain data through the Fast Fourier transform (FFT).

### 2.4 Transgenic/ nontransgenic DNA test

In order to explore the relationship between experimental phenomenon and the microscopic mechanism, transgenic/nontransgenic DNA test was carried out in this research. Transgenic/nontransgenic DNA was obtained from Department of Agricultural and Biotechnology at Beijing Forestry University. The supplied DNA was dissolved in NaCl prior to lyophilization of the sample, implying that the counterion is Na. Polyethylene samples were also measured as comparison and reference. Each sample was removed from the buffer just prior to measurement, and excess buffer was allowed to drip off the tissue by tilting the polyethylene plate. The tissue was then mounted vertically at the THz focal point with the THz beam directed through the polyethylene plates. All experiments were performed in triplicate. The average value of total 6 measurements ± standard deviation was regarded as the final results.

### 2.5 Data analysis

The THz optical refractive index of the cotton seeds is calculated using standard algorithm (Fischer et al., 2005; Heavens, 1992):

(1) $$n\left(f\right)=\frac{|\varphi_{s}\left(f\right)‐\varphi_{\text{ref}}\left(f\right)|c}{2\pi fd}+1$$

where φ_s_(*f*) and φ_ref_ (*f*) are the phase angles of the Fourier transforms of the powder transmissions of the seed sample, *I_s_* is the amplitude values of powder transmissions of the samples, and *I*_ref_ is the amplitude values of powder transmissions of the reference (the blank polyethylene chemicals), and *c* is the light speed and *f* is the frequency. *d* is the optical path the THz pulse of radiation passing through samples before being detected. To derive the dielectric constant, the extinction coefficient, *β(f)*, is obtained as

(2) $$\beta \left(f\right)=\ln\left[\frac{4n(f)}{\rho \left(f\right){\left[n\left(f\right)+1\right]}^{2}}\right]\frac{c}{2\pi fd}$$

Where *ρ(f)* is the amplitude ratio of the Fourier transforms of *I_s_* and *I*_ref_. The absorption coefficient of sample, *α*, is deducted as

(3) $$\alpha \left(f\right)=\frac{4\pi f\beta (f)}{c}$$

SPSS software package was used for statistical analysis (version 13, SPSS).

## 3 RESULTS AND DISCUSSION

### 3.1 Preparing technology optimization

**Figure 2:**
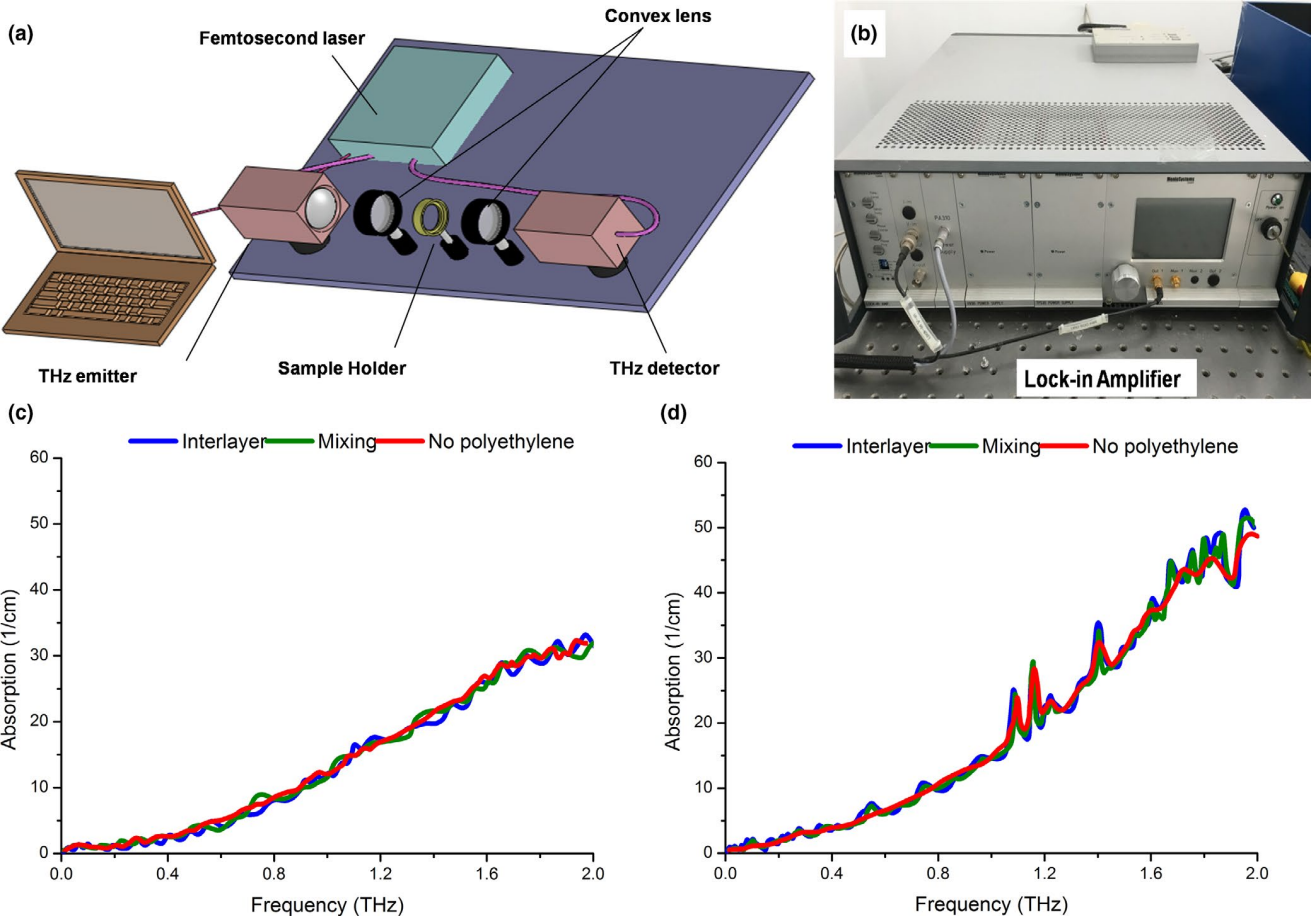
Experimental setup for the THz analysis process (a) and the lock‐in amplifier (b). THz absorption spectrogram of nontransgenic (c) and transgenic (d) cotton seeds using interlayer/mixing polyethylene substance or not

Terahertz waves are capable of easily propagating through most of the dielectric materials (such as plastics, clothing and packaging) and even through some construction materials, which can be analyzed rapidly. The experimental setup was shown in Figure 2a,b and the samples should not to be touched which avoids any damages in the detection process. It is reported that polyethylene is almost transparent at THz region compared to other bands in the whole electromagnetic wave (Bruni, Conti, Corvi, Rocchi, & Tosi, 2002; Palka, Panowicz, Ospald, & Beigang, 2015); however, most measurements were made compared to a sample consisting solely and fully mixed with polyethylene. In this study, we investigated numerous samples formation methods using commercially standard polyethylenes. As shown in Figure 2c,d, there were small fluctuations in the THz absorption spectrogram due to polyethylene chemical powder, but the general tendency was more or less the same. After the phase's error correction, the absorption index of cotton seeds was calculated and compared with that of the polyethylene pellet, which did not have typical absorption features in 0.1–2.0 THz band. It was found that the minor fluctuation errors to determine the measurement position of samples may be caused either by sample–projectile interactions or by making inhomogeneity of the samples. Moreover, the group refractive index of the thinner samples at a single point was calculated and also showed typical fluctuates (Hall & Richard, 1967).

Polyethylene is widely used in versatile applications, and it is used as medical polymers in contact with living tissues or biological fluids (Exter, Fattinger, & Grischkowsky, 1989; Tomita, Kitakura, Onmori, Ikada, & Aoyama, 1999). In THz band, polyethylene also showed very weak absorption features which made it a good reference material. The transmission spectrum was normalized to the spectrum of a pure polyethylene film. This distinguishing method was in a nondestructive and contactless manner. The nondestructive method for inspecting inner polyethylene is, therefore, beneficial for the quality control of polyethylene as source materials. Polyethylene also shows stiff enough to format the tissue into tablets, and pliable enough for scissors to cut conveniently. From the above analysis and exponential exploration, the obtained results reflect the samples’ real conditions very close and interlayer polyethylene seems to be a good choice for the THz technique.

### 3.2 Transgenic and nontransgenic data analysis

**Figure 3:**
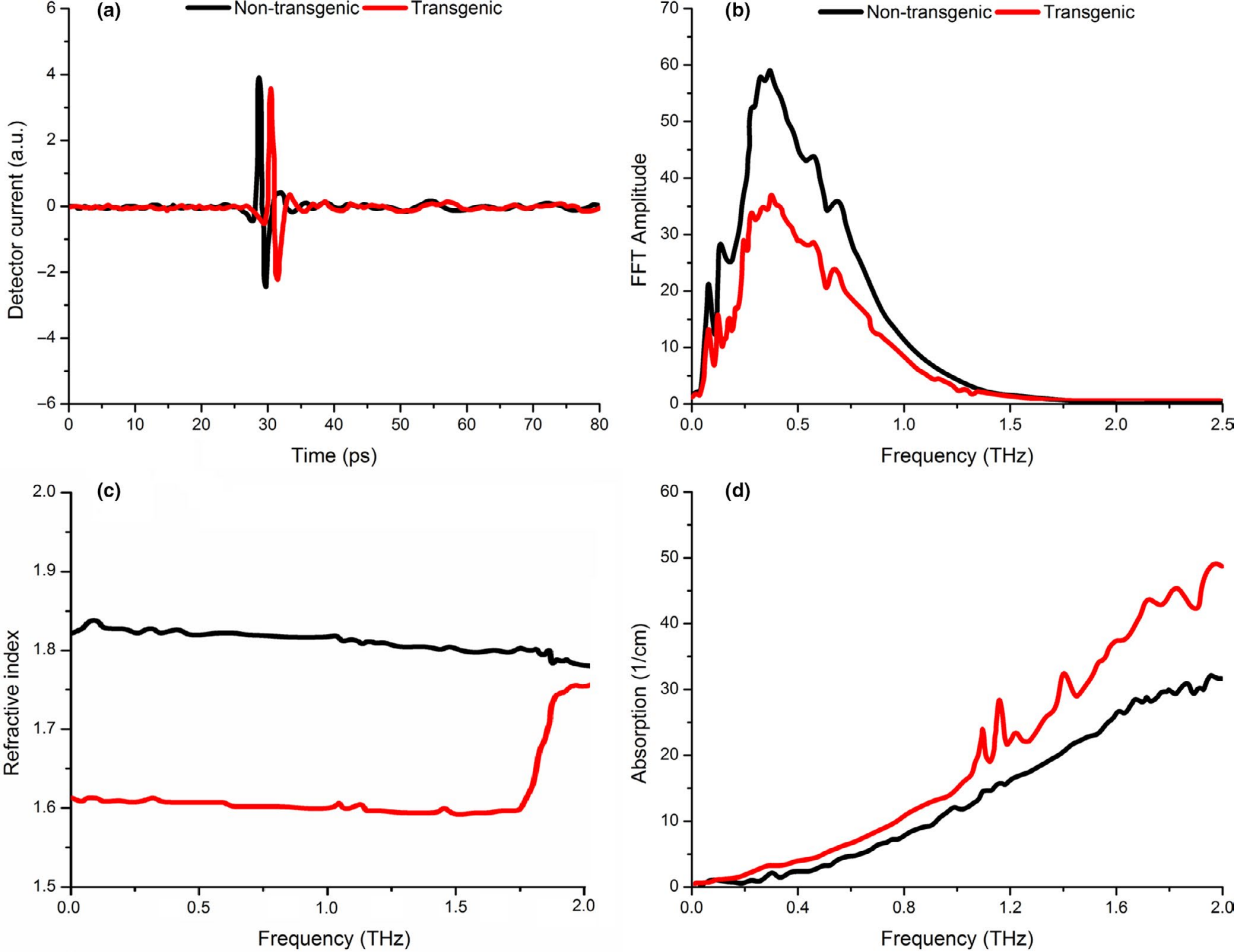
THz time‐domain spectra (a), frequency‐domain spectra (b), THz refractive spectrogram (c) and THz absorption spectrogram (d) of nontransgenic (black line) and transgenic (red line) cotton seeds

From the measured THz data, the average amplitudes and phases of transgenic and nontransgenic cotton seeds showed very close detector current in THz time‐domain spectroscopy (Figure 3a). The curves showed only minor difference of the waveforms and the pulse amplitudes of nontransgenic and transgenic cotton seeds. So it is unobvious to discriminate transgenic and nontransgenic cotton seeds just using time‐domain spectra and other visual angles should be explored. The amplitude of different cotton seeds was then calculated under the frequency‐domain transformed from time‐domain data (Figure 3b). The measured signals from samples using THz equipment usually contain not only the initial pulses which were transmitted through the seeds, but also numerous subsequent pulses of delay time generated by internal reflections. Refractive index of the transgenic cotton seeds was lower than the nontransgenic ones (Figure 3c). The step change of reflective index around 1.6 THz reflects THz phase changes of samples and it may be caused by three reasons: one is the higher oil content; the second is a new ingredient due to DNA of transgenic samples, and the third is that the phase in Equation (1) is determined within 2*πK*, where *K* is integer.

The absorption curves of transgenic and nontransgenic cotton seeds are shown in Figure 3d. The spectroscopy curves of two varieties showed similar tendency; however, for the transgenic cotton seeds, there were significant peaks in 1.0–1.2 and 1.3–1.5 THz region in details. On the contrary, there were no distinctive peaks for the nontransgenic cotton seeds in these THz regions. As for cotton bio‐samples, the intermolecular interactions, such as van der Waals forces, hydrogen bonding, usually modify the intramolecular vibrations’ mode structure and cause additional vibrational modes including collective dynamics of certain molecules (Brucherseifer et al., 2000). The intramolecular interactions are usually much more obvious than intermolecular interactions, and the fingerprints of intermolecular modes often emerge in THz band. In addition, the results also showed that cotton seed samples in nanoparticle scale can provide important information in THz band and the nanoparticle scale size fitted this study well.

### 3.3 DNA analysis

**Figure 4:**
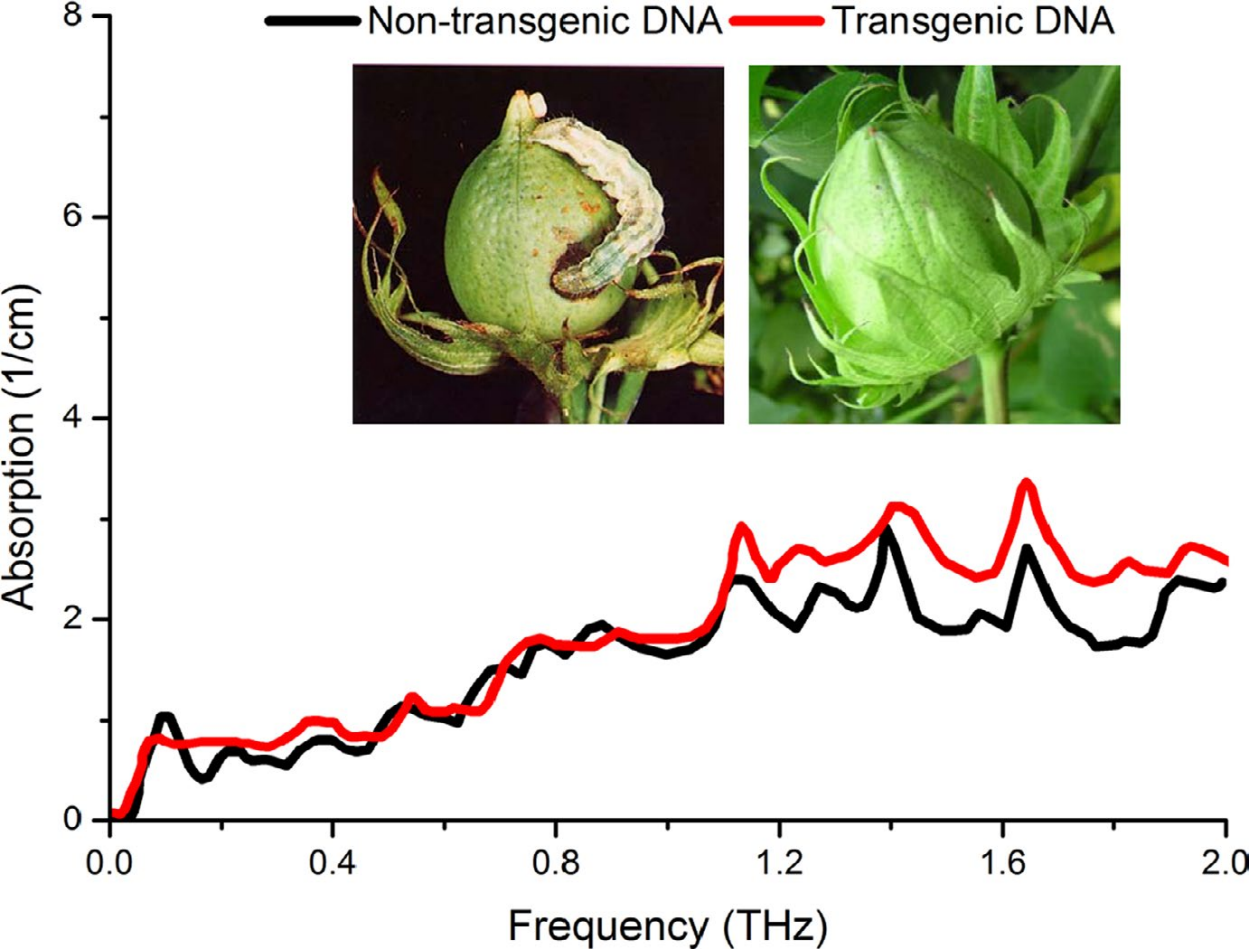
THz absorption spectrogram of nontransgenic (black line) and transgenic (red line) DNA

The molecule's global conformation is very important in typical bio‐molecules such as DNA proteins and RNA. Although previous calculations are not accurate enough for the possible influences of van der Waals, the observations of interhelical excitations indicate THz might be a useful tool for label‐free detection of binding state of DNA (Brucherseifer et al., 2000). As shown in Figure 4, transgenic DNA had higher absorption index than nontransgenic DNA. In particularly, from 1.0 to 1.6 THz region, there were 3–4 peaks that corresponded to the cotton seed samples (Figure 3d) (Zhang et al., 2016). Due to the minor inhomogeneities during the macroscopic sample‐making process including grinding, mixing and pressing, and the environmental influences, the absolute value of absorption coefficient varied slightly (about 10%) in the actual measurements. The presented index of THz absorption spectrogram is an average one of the sample materials.

Based on the presented achievements, the refraction index of DNA molecules was paid much more attention recently by Brucherseifer who employed the far‐infrared parameters of DNA samples to study the hybridization state (Fedotov et al., 2015). However, different results in the observed transitions indicate the actual difficulties met in reliably analyzing DNA using the above technologies, as shown by recent analysis of hybridized DNA in time‐domain under different humidity levels. After comparison with similar spectral features of molecules containing similar side groups (Fischer et al., 2002), we tentatively interpret these additional lines as vibrational signatures associated with the sugar groups attached to the nucleobases.

The presented measurements represent an observation of binding‐state‐dependent properties of polynucleotides in THz band. This observation does not only raise a probing concept for the essential analysis of bio‐molecules which can rapidly conduct dynamic conformation analysis, but also potentially advance the basis for the development of future THz‐based gene probing techniques.

## 4 CONCLUSIONS

In conclusion, the presented preliminary study has designed to obtain and observe the absorption difference between the transgenic and nontransgenic cotton seeds in THz band and tried to explore a method to identify the transgenic cotton seeds from its counterparts using THz spectroscopy probing methods, which demonstrates the capability of THz techniques for detecting the binding state of genetic material directly. From the study, it was found that polyethylene chemical and the proposed sample‐making method are good choices for the THz technique, which made the results obtained in this investigation closely reflecting the real conditions. The results showed cotton seeds samples in nanoparticle‐scale can provide important information in THz band and there were significant peaks in 1.0–1.2 and 1.3–1.5 THz region for the transgenic cotton seeds. From 1.0 to 1.6 THz region, transgenic DNA had higher absorption index than nontransgenic DNA which corresponded to the cotton seed samples well. The response of THz absorption spectrogram changes to transgenic and nontransgenic cotton seeds can provide a theoretical basis for further research to distinguish GM organisms from crop varieties.

## CONFLICT OF INTEREST

The authors declare that they do not have any conflict of interest.

## ETHICAL APPROVAL AND CONSENT TO PARTICIPATE

This study does not involve any human or animal testing. The manuscript was verified and approved by the coauthor listed for publication.

## HUMAN AND ANIMAL RIGHTS

The study reported here did not require treatments for humans or animals; thus, human and animal rights concerns do not apply.

## References

[fsn31846-bib-0001] Ali, A. , Ahmed, S. , Nasir, I. A. , Rao, A. Q. , Ahmad, S. , & Husnain, T. (2016). Evaluation of two cotton varieties CRSP1 and CRSP2 for genetic transformation efficiency, expression of transgenes Cry1Ac+ Cry2A, GT gene and insect mortality. Biotechnology Reports, 9, 66–73. 10.1016/j.btre.2016.01.001 28352594 PMC5360982

[fsn31846-bib-0002] And, J. T. K. , & Schmuttenmaer, C. A. (1996). Far‐infrared dielectric properties of polar liquids probed by femtosecond terahertz pulse spectroscopy†. Journal of Physical Chemistry, 100, 10373–10379.

[fsn31846-bib-0003] Aragao, F. J. , Vianna, G. R. , Carvalheira, S. B. , & Rech, E. L. (2005). Germ line genetic transformation in cotton (*Gossypium hirsutum* L.) by selection of transgenic meristematic cells with a herbicide molecule. Plant Science, 168, 1227–1233. 10.1016/j.plantsci.2004.12.024

[fsn31846-bib-0004] Bakshi, A. (2003). Potential adverse health effects of genetically modified crops. Journal of Toxicology and Environmental Health Part B, Critical Reviews, 6, 211–226. 10.1080/10937400306469 12746139

[fsn31846-bib-0005] Baranski, R. , & Baranska, M. (2008). Discrimination between nongenetically modified (Non‐GM) and GM plant tissue expressing cysteine‐rich polypeptide using FT‐raman spectroscopy. Journal of Agricultural & Food Chemistry, 56, 4491–4496. 10.1021/jf800410m 18494491

[fsn31846-bib-0006] Brucherseifer, M. , Nagel, M. , Bolivar, P. H. , Kurz, H. , Bosserhoff, A. , & Büttner, R. (2000). Label‐free probing of the binding state of DNA by time‐domain terahertz sensing. Applied Physics Letters, 77, 4049–4051. 10.1063/1.1332415

[fsn31846-bib-0007] Bruni, P. , Conti, C. , Corvi, A. , Rocchi, M. , & Tosi, G. (2002). Damaged polyethylene acetabular cups microscopy FT‐IR and mechanical determinations. Vibrational Spectroscopy, 29, 103–107. 10.1016/S0924-2031(01)00193-X

[fsn31846-bib-0008] Exter, M. V. , Fattinger, C. , & Grischkowsky, D. (1989). Terahertz time‐domain spectroscopy of water vapor. Optics Letters, 14, 1128–1130. 10.1364/OL.14.001128 19753077

[fsn31846-bib-0009] Fedotov, V. A. , Wallauer, J. , Walther, M. , Perino, M. , Papasimakis, N. , & Zheludev, N. I. (2015). Wavevector selective metasurfaces and tunnel vision filters. Light Science & Applications, 4, 1–6. 10.1038/lsa.2015.79

[fsn31846-bib-0010] Fischer, B. M. , Hoffmann, M. , Helm, H. , Wilk, R. , Rutz, F. , Kleine‐Ostmann, T. , … Jepsen, P. U. (2005). Terahertz time‐domain spectroscopy and imaging of artificial RNA. Optics Express, 13, 5205–5215. 10.1364/OPEX.13.005205 19498511

[fsn31846-bib-0011] Fischer, B. M. , Walther, M. , & Jepsen, P. U. (2002). Far‐infrared vibrational modes of DNA components studied by terahertz time‐domain spectroscopy. Physics in Medicine & Biology, 47, 3807–3814. 10.1088/0031-9155/47/21/319 12452571

[fsn31846-bib-0012] Hall, R. T. , & Dowling, J. M. (1967). Pure rotational spectrum of water vapor. Journal of Chemical Physics, 47(7), 2454 10.1063/1.1703330

[fsn31846-bib-0020] Heavens, O. S. (1992). Handbook of Optical Constants of Solids II. Journal of Modern Optics, 39(1), 189–189. 10.1080/716099804a

[fsn31846-bib-0013] Jiao, Z. , Si, X. , Li, G. , Zhang, Z. , & Xu, X. (2010). Unintended compositional changes in transgenic rice seeds (*Oryza sativa* L.) studied by spectral and chromatographic analysis coupled with chemometrics methods. Journal of Agricultural & Food Chemistry, 58, 1746–1754. 20050687 10.1021/jf902676y

[fsn31846-bib-0014] Leimanis, S. , Hernández, M. , Fernández, S. , Boyer, F. , Burns, M. , Bruderer, S. , … Remacle, J. (2006). A microarray‐based detection system for genetically modified (GM) food ingredients. Plant Molecular Biology, 61, 123–139. 10.1007/s11103-005-6173-4 16786296

[fsn31846-bib-0015] Li, C. , Li, B. , & Ye, D. (2020). Analysis and identification of rice adulteration using terahertz spectroscopy and pattern recognition algorithms. IEEE Access, 8, 26839–26850. 10.1109/ACCESS.2020.2970868

[fsn31846-bib-0016] Liu, W. , Liu, C. , Hu, X. , Yang, J. , & Zheng, L. (2016). Application of terahertz spectroscopy imaging for discrimination of transgenic rice seeds with chemometrics[J]. Food Chemistry, 210, 415–421. 10.1016/j.foodchem.2016.04.117 27211665

[fsn31846-bib-0017] Lois, C. , Hong, E. J. , Pease, S. , Brown, E. J. , & Baltimore, D. (2002). Germline transmission and tissue‐specific expression of transgenes delivered by lentiviral vectors. Science, 295, 868–872. 10.1126/science.1067081 11786607

[fsn31846-bib-0019] Nesvold, H. , Kristoffersen, A. B. , Holst‐Jensen, A. , & Berdal, K. G. (2005). Design of a DNA chip for detection of unknown genetically modified organisms (GMOs). Bioinformatics, 21, 1917–1926. 10.1093/bioinformatics/bti248 15647302

[fsn31846-bib-0021] Palka, N. , Panowicz, R. , Ospald, F. , & Beigang, R. (2015). 3D Non‐destructive imaging of punctures in polyethylene composite armor by THz time domain spectroscopy. Journal of Infrared, Millimeter, and Terahertz Waves, 36, 770–788. 10.1007/s10762-015-0174-4

[fsn31846-bib-0022] Tomita, N. , Kitakura, T. , Onmori, N. , Ikada, Y. , & Aoyama, E. (1999). Prevention of fatigue cracks in ultrahigh molecular weight polyethylene joint components by the addition of vitamin E. Journal of Biomedical Materials Research Part A, 48, 474–478. 10.1002/(SICI)1097-4636(1999)48:4<474:AID-JBM11>3.0.CO;2-T 10421689

[fsn31846-bib-0023] Tonouchi, M. (2007). Cutting‐edge terahertz technology. Nature Photonics, 1, 97–105. 10.1038/nphoton.2007.3

[fsn31846-bib-0024] Vaïtilingom, M. , Pijnenburg, H. , Gendre, F. , & Brignon, P. (1999). Real‐time quantitative PCR detection of genetically modified Maximizer maize and Roundup Ready soybean in some representative foods. Journal of Agricultural & Food Chemistry, 47, 5261–5266. 10.1021/jf981208v 10606606

[fsn31846-bib-0027] Wietzke, S. , Jansen, C. , Reuter, M. , Jung, T. , Hehl, J. , Kraft, D. … Koch, M. (2010). Thermomorphological study of the terahertz lattice modes in polyvinylidene fluoride and high‐density polyethylene. Applied Physics Letters, 97, 022901–022903. 10.1063/1.3462312

[fsn31846-bib-0025] Xie, L. , Ying, Y. , Ying, T. , Yu, H. , & Fu, X. (2007). Discrimination of transgenic tomatoes based on visible/near‐infrared spectra. Analytica Chimica Acta, 584, 379–384. 10.1016/j.aca.2006.11.071 17386628

[fsn31846-bib-0026] Xu, L. , Li, B. , Zuo, J. , & Zhang, C. (2015). The effect of different particle sizes of polyethylene on the absorption of myrrh in mixtures. 2015 International Conference on Optical Instruments and Technology: Terahertz Technologies and Applications, 9625, 96250D1–96250D7

[fsn31846-bib-0028] Xu, W. , Liu, X. , Xie, L. , & Ying, Y. (2014). Comparison of fourier transform near‐infrared, visible near‐infrared, mid‐infrared, and raman spectroscopy as non‐invasive tools for transgenic rice discrimination. Transactions of the Asabe, 57, 141–150.

[fsn31846-bib-0029] Zhang, F. , Wang, H. W. , Tominaga, K. , Hayashi, M. , Hasunuma, T. , & Kondo, A. (2017). Application of THz vibrational spectroscopy to molecular characterization and the theoretical fundamentals: An illustration using saccharide molecules. Chemistry ‐ An Asian Journal, 12, 324–331. 10.1002/asia.201601419 27883277

[fsn31846-bib-0030] Zhao, Y. , Qian, Q. , Wang, H.‐Z. , & Huang, D. (2007). Co‐transformation of gene expression cassettes via particle bombardment to generate safe transgenic plant without any unwanted DNA. Vitro Cellular & Developmental Biology‐Plant, 43, 328–334. 10.1007/s11627-007-9051-8

